# Hip fracture in hospitalized medical patients

**DOI:** 10.1186/1471-2474-14-15

**Published:** 2013-01-08

**Authors:** Antonio Zapatero, Raquel Barba, Jesús Canora, Juan E Losa, Susana Plaza, Jesús San Roman, Javier Marco

**Affiliations:** 1 Servicio de Medicina Interna, Hospital Universitario de Fuenlabrada, Fuenlabrada, Madrid, Spain; 2 Servicio de Medicina Interna, Hospital Rey Juan Carlos, Móstoles, Madrid, Spain; 3 Servicio de Medicina Interna, Hospital Universitario Fundación Alcorcón, Alcorcón, Madrid, Spain; 4 Servicio de Medicina Interna, Hospital Severo Ochoa, Leganes, Madrid, Spain; 5 Departamento de Medicina y Cirugía, Universidad Rey Juan Carlos, Madrid, Spain; 6 Servicio de Medicina Interna, Hospital Clínico San Carlos, Madrid, Spain

**Keywords:** Hip-fracture, Hospitalized, Internal medicine, Morbidity, Mortality, Security

## Abstract

**Background:**

The aim of the present study is to analyze the incidence of hip fracture as a complication of admissions to internal medicine units in Spain.

**Methods:**

We analyzed the clinical data of 2,134,363 adults who had been admitted to internal medicine wards. The main outcome was a diagnosis of hip fracture during hospitalization.

Outcome measures included rates of in-hospital fractures, length of stay and cost.

**Results:**

A total of 1127 (0.057%) admittances were coded with an in-hospital hip fracture. In hospital mortality rate was 27.9% vs 9.4%; p < 0.001, and the mean length of stay was significantly longer for patients with a hip fracture (20.7 days vs 9.8 days; p < 0.001). Cost were higher in hip-fracture patients (6927€ per hospitalization vs 3730€ in non fracture patients). Risk factors related to fracture were: increasing age by 10 years increments (OR 2.32 95% CI 2.11-2.56), female gender (OR 1.22 95% CI 1.08-1.37), admission from nursing home (OR 1.65 95% CI 1.27-2.12), dementia (1.55 OR 95% CI1.30-1.84), malnutrition (OR 2.50 95% CI 1.88-3.32), delirium (OR 1.57 95% CI 1.16-2.14), and anemia (OR 1.30 95%CI 1.12-1.49).

**Conclusions:**

In-hospital hip fracture notably increased mortality during hospitalization, doubling the mean length of stay and mean cost of admission. These are reasons enough to stress the importance of designing and applying multidisciplinary plans focused on reducing the incidence of hip fractures in hospitalized patients.

## Background

The incidence of hip fracture is increasing throughout the world, due among other reasons to the progressive aging of the population, with the number of people over 65 expected to double in the next three decades [1]. The raw incidence of hip fracture in Spain is 511 cases/100,000/year in patients age 65 or older [2]. Moreover, demographic studies show that hip fractures are more common in institutionalized patients [3] and in patients hospitalized for another cause, in whom the risk can be up to 11 times greater [4].

When hip fracture occurs as a complication of hospital admission, it can have devastating effects on the patient, producing clinical and psychological consequences, prolonging hospital stay, increasing mortality and notably raising the cost of hospital care [3,5]. Hip fracture in hospitalized patients has been related to a series of predisposing factors, particularly falls in the hospital [6]. They are more frequent in medical than in surgical wards [7,8]. In-hospital falls are considered a quality-of-care indicator [9,10]. Hip fracture in postsurgical patients was included as an indicator of complications due to the care process during hospitalization by Lezzoni et al [11]. in 1992, when they developed the Complications Screening Program (CSP). Hip fracture was later listed as one of the Patient Safety Indicators by the Agency for Healthcare Research and Quality [12] and as a safety indicator in the OECD HCQI Project (Organisation for Economic Cooperation and development Health Care Quality Indicators Project) [13].

Patients admitted to internal medicine units have many of the factors associated with increased risk of falls and fractures during admission such as advanced age, mobility and cognitive impairment, confusional syndrome development during admission, polimedication and a high demand for nursing care [6].

The aim of the present study by the Internal Medicine Spanish Society Management Group was to analyze the incidence of hip fracture as a complication of admission to internal medicine units and to describe their epidemiological characteristics, including associated risk factors, hospital course, mortality, and impact on hospital stay and cost of care.

## Methods

We identified every patient discharged from the Internal Medicine departments of hospitals in the Spanish Public Health Service between January 1st, 2005 and December 31st, 2008. Hospital discharge data were obtained from the Basic Minimum Data Set (BMDS). This BMDS registry is compulsory for every patient admitted to a hospital of the Spanish National Health Service, a system that cares for more than 90% of the country population. All centres submit this information to the Spanish Health Ministry. BMDS contains socio-demographic and clinical data for each documented hospital discharge including: gender and age, primary and secondary diagnoses [according to the International Classification of Diseases, Ninth Revision, Clinical Modification (ICD-9-CM) code]; primary and secondary procedures; discharge status; length of stay; and hospital characteristics (less than 200 beds; 200 to 500 beds; 500 to 1,000 beds; more than 1,000 beds). For every patient, a diagnosis-related group (DRG) was identified. DRGs are a way of classifying patient hospitalizations by diagnosis and procedure on the assumption that similar costs are expended on patients by using similar resources. Each DRG has a relative weight that reflects the intensity of resources consumed. Emergency admissions are those produced directly from the Emergency Department. Permission was obtained for using the data.

The BMDS assigns a main diagnosis related to cause of admission to hospital. Besides, it is possible to add up to 12 secondary diagnosis including complications occurring during hospitalization, recurring of previous diseases or new diagnosis arising from studies performed during admission. The main diagnosis should always be related with the main symptom initiating admission following ICD-9-CM coding system. That is the reason why a patient with hip fracture will have a main diagnosis of hip fracture and a patient with a hip fracture occurring during admission will have hip fracture as a secondary diagnosis.

Inclusion criteria: patients admitted to an Internal Medicine department between January 1st, 2005 and December 31st, 2008, without a principal diagnosis of hip fracture (ICD-9-CM 820.00-820.22; 820.30-820.32; 820.8; 820.9). The main outcome was a diagnosis of hip fracture during admission. Patients with a hip fracture at admission were excluded. The indicator was defined based on the Agency for Healthcare Research and Quality (AHRQ) patient safety indicators as cases on in-hospital hip fracture per 100 medical discharges [14]: numerator discharges with an ICD-9-CM code for hip fracture in any secondary diagnosis field (820.00-820.22; 820.30-820.32; 820.8; 820.9) among cases meeting the inclusion and exclusion rules for the denominator; denominator all medical discharges age 18 and older. Excluded cases with principal diagnosis of hip fracture, cases with diseases and disorder of the musculoskeletal system and connective tissue (MCD 8), cases with principal diagnosis of seizure, syncope, stroke, coma, cardiac arrest, poisoning, trauma, delirium and other psychoses or anoxic brain injury, cases with diagnosis of metastatic cancer, lymphoid malignancy or bone malignancy or self-inflicted injury, discharges in MDC 8 (disease and disorders of the musculoskeletal system and connective tissue) and MCD 14 (pregnancy, childbirth an puerperium) [15].

The Age Adjusted Charlson Co-morbidity Index (CCI) was computed for each patient. This index reflects the number and importance of comorbid diseases, relies on ICD-9-CM categories, and was used to adequately adjust for severity of illness [16,17].

The following risk factors for hip fracture were identified using ICD-9-CM codes in any secondary diagnosis field: Cardiac Disease: ICD-9-CM:398.91, 404, 402.11, 402.91,428-428.9, Dementia: ICD-9-CM: 290–290.9, Delirium ICD-9-CM: 298.9, 293.0, 293.9,0293.8, Chronic pulmonary disease ICD-9-CM: 490–496, 500–505, 506.4, Cancer ICD-9-CM:140.0-172.9,174.0-195.8, 200–208.9, V10.0-V10.9, Metastasic cancer: ICD-9-CM:196.0-199.99, Cerebrovascular disease ICD-9-CM: 430438, Malnutrition: ICD-9-CM: 260–263.9, Diabetes: ICD-9-CM: 250.00-250.99, Myocardial infarction: ICD-9-CM: 410–410.9, Chronic renal failure ICD-9-CM: 585586.99, 582.0-582.9, 583.0-583.7, 588.0-588.9 and Anaemia: ICD-9-CM: 280.00-285.99.

Hospitalization cost estimation in Spain has been developed by the Spanish Ministry of Health and it is basically based on DRG coding system. DRGs are a way of classifying patient hospitalisations by diagnosis and procedure on the assumption that similar costs (direct, indirect and structural), are expended on patients allocated similar resources [18].

### Data analysis

Differences in the distribution of various demographic and clinical characteristics between patients who presented hip fracture during hospitalization and those who did not, were examined. We used the chi-square test for categorical variables with the Yates correction, the Fisher’s exact test for dichotomic variables when the expected value of a cell was less than 5, and Student T for quantitative variables (difference of means). The unadjusted Odds-Ratios (OR) and 95% Confidence Intervals (CI) were estimated from the logistic regression coefficients. The most clinically relevant variables and those with statistical significance (p < 0.1) in the univariate analyses of every subgroup were introduced in the logistic regression analysis, to determine independent risk factors for hip fracture during hospitalization. A logistic regression analysis with backward stepwise procedure and p > 0.10 as the criterion for exclusion was used to find the variables independently associated with hip fracture during admission. Effects of in-hospital length of stay and medical cost have been adjusted for age, gender, comorbidities and potential confounders (anemia, dementia, delirium) by linear regression models.

All statistical analysis was carried out with the use of a SPSS Software version 16.

## Results

Between January 2005 and December 2008 the dataset included 2,134,363 admissions. Of these, 1,991,911 (93.3%) met the basic inclusion criteria. A total of 1127 (0.057%) were coded with an in-hospital hip fracture. The mean age of in-hospital hip fracture patients was 81.52 years (SD 10.15) and 57.2% were women. An age-adjusted CCI score >2 was present in 1080 (95.8%) of cases.

The hospitalization cost for patients who developed hip fracture during admittance was 6927 € (SD 5572), 3138 € higher than the mean cost of an admitted patient in Internal Medicine (3789 € [SD 2450]) [18]. The general characteristics of all hospital discharges are summarised in Table 1.

**Table 1:** Demographic and clinical details for hospital episodes associated with in hospital hip fractures

	Patients with in-hospital hip fracture*	**Patients without in-hospital hip fracture**	**p**
**N (admissions)**	1127	1,989,784	
**Age (years), SD (mean)**	81.52 (10.35)	71.33 (16.76)	<0.001
**Gender (male, %)**	482 (42.8)	1,0614,39 (57.2)	<0.001
**Admission from nursing home (%)**	64 (5.7)	42987 (2.2)	<0.001
**Length of stay (days), SD (mean)**	20.7(25.34)	9.8 (11.20)	<0.001
**Death during admission (%)**	314 (27.9)	187535 (9.4)	<0.001
**Discharge to rehabilitation hospital (%)**	116 (10.3)	69544 (3.5)	<0.001
**Cost of hospital admission (€), SD (mean)**	6927€ (5572)	3730€ (2344)	<0.001
**Charlson index > 2 (%)**	1080 (95.8)	1,610,996 (81)	<0.001
**Cancer (%)**	106 (9.4)	198,277 (10.0)	0.548
**Metastasic cancer (%)**	30 (2.7)	56783 (2.9)	0.778
**Chronic heart failure (%)**	248 (22.0)	454,746 (22.9)	0.522
**Chronic cerebrovascular disease (%)**	110 (9.8)	158,020 (7.9)	0.025
**Chronic pulmonary disease (%)**	253 (22.4)	513,387 (25.8)	0.012
**Dementia (%)**	155 (13.8)	116612 (5.9)	<0.001
**Diabetes (%)**	275 (24.4)	526,951 (26.5)	0.114
**Myocardial infarction (%)**	102 (9.1)	188,634 (9.5)	0.643
**Chronic renal disease (%)**	94 (8.3)	143,242 (7.2)	0.147
**Delirium (%)**	43 (3.8)	36248 (1.8)	<0.001
**Malnutrition (%)**	51 (4.5)	29695 (1.5)	<0.001
**Anemia (%)**	247 (21.9)	305,865 (15.4)	<0.001

*Internal Medicine discharges with ICD-9-CM code for fracture in any secondary diagnosis field, without a principal diagnosis of Hip fracture.

Over 97.6% of episodes were for individuals over 50 years of age (Table 2). The most prevalent comorbidities among the in-hospital hip fracture population were: diabetes (24.4%), pulmonary disease (22.4%), chronic heart failure (22%), anemia (21.9%) and dementia (13.8%) (Table 1).

**Table 2:** Prevalence of in-hospital hip fracture related to age

**Age**	**Patients**	**In-hospital hip fracture**	**Cases/100 patients**	**Cases/10**^**4**^patients · day*
**20-29**	60713	1	0.0165	0.028
**30-39**	87684	7	0.079	0.098
**40-49**	120270	19	0.158	0.168
**50-59**	155988	22	0.141	0.152
**60-69**	268437	70	0.261	0.274
**70-79**	593423	286	0.482	0.472
**80-89**	577053	535	0.928	0.845
**>90**	125089	187	1.495	1.303

*The incidence rate of in-hospital hip fracture per 10 [4] patient-days.

Malnutrition and dementia were the major comorbid conditions associated with in-hospital hip fracture (4.5% vs 1.5%; OR 3.13 95%CI 2.364.14, and 13.8% vs 5.9%; OR 2.56 95%CI 2.16-3.03; p < 0.001 for both). There were also a higher proportion of episodes with a comorbid diagnosis of delirium among in-hospital hip fracture patients, compared with patients without in-hospital hip fracture (3.8% vs 1.8%; p < 0.001). Anemia (21.9% vs 15.4%; p < 0.001), and chronic cerebrovascular disease (9.8% vs 7.9%; p = 0.02) were also associated to in-hospital hip fracture.

The mean length of stay was longer for episodes associated with a fracture (20.7 days vs 9.8 days; p < 0.001). Also the mean cost was higher in in-hospital hip fracture (3730 vs 6927 €). When these variables were adjusted by potential confounders, the differences remained statistically significant (20.6 vs 9.7 days and 3730 vs 6894 €). Hospital characteristics of these patients are summarised in Table 1. Emergency admissions accounted for most of the episodes in which fracture occurred (88.2%), although the risk of hip-fracture was higher in elective admissions (OR 1.63 95%CI 1.36-1.95). Admission from nursing homes accounted for the highest volume of episodes, with a double risk for hip-fracture than home-admissions (OR 2.73 95%CI 2.11-3.50).

A total of 314 (27.9%) patients with in-hospital hip fracture died during hospitalization. Dying patients were older, 84.2 years (SD 8.3) vs 80.1 years (SD 10.8); p < 0.001, and had a higher co-morbidity (CCI index > 2, 97.4% vs. 94.7%; p = 0.002). The mortality risk was more than double in patients with hip-fracture after adjustment for age, gender and comorbidities (OR 2.66 CI 95% 2.34-3.02).

In the logistic regression model the variables showing a strongest associations with hip-fracture were: increasing age (OR 2.32 95% CI 2.11-2.56), female gender (OR 1.22 95% CI 1.081.37), admissions from nursing home (OR 1.65 95% CI 1.27-2.12), dementia (1.55 OR 95% CI 1.30-1.84), malnutrition (OR 2.50 95% CI 1.87-3.32), delirium (OR 1.57 95% CI 1.162.14), and anemia (OR 1.30 95% CI 1.12-1.49) (Table 3).

**Table 3:** Risk factors of in-hospital hip fracture

	**OR**	**Lower 95%CI**	**Upper 95%CI**	**P**
**Female gender**	1.22	1.08	1.37	<0.001
**Age (10 year increase)**	2.32	2.11	2.56	<0.001
**Admission from nursing home**	1.65	1.27	2.12	<0.001
**Malnutrition**	2.50	1.88	3.32	<0.001
**Delirium**	1.57	1.16	2.14	0.004
**Dementia**	1.55	1.30	1.84	<0.001
**Anemia**	1.30	1.12	1.49	<0.001

OR: odds ratio. CI: confidence interval.

Mutivariable analyses.

## Discussion

After analyzing more than 2 million admissions, we found an incidence of hip fracture in patients admitted to internal medicine of 0.58 per 1000 admissions. In a Spanish study of postsurgical patients using the same methodology, the rate of hip fracture was 0.1 per 1000 patients operated upon [15]. Therefore, hip fracture as a complication of admission is almost 6 times more frequent in patients admitted to internal medicine than in those recovering from surgery. The risk factors associated with this complication were age, female sex, dementia, confusional syndrome, malnutrition, comorbidity and admission from a nursing home. The most evident consequences of hip fracture were a two-fold increase in hospital stay, a three-fold increase in mortality and a twofold increased in cost per admission compared to patients without hip fracture.

Studies by other authors had already underlined the relevance of hip fracture as a complication in postoperative patients. In a study made in England, Raleigh et al [19] report that hip fracture in postoperative patients produces an excess in hospital stay of 17.09 days and an excess in mortality of 18.20%. Brand et al [20] reviewed the administrative data of public hospitals of the state of Victoria, Australia, for a period of 10 years. They report an incidence of hip fracture in hospitalized patients of 0.14%, which remained stable for the duration of the study. The presence of hip fracture was associated with a 30% increase in mortality and a fourfold increase in hospital stay. Johal et al [5] demonstrated that the mortality of patients with hip fracture occurring in the hospital was 18% at 30 days and 47% after 1 year, twice as high as the mortality of patients with hip fracture occurring in the community. Most in-hospital hip fractures occurred in medical and geriatric units, in fragile patients with cognitive impairment and a high incidence of comorbidities, as is the case in our series.

Following the aforementioned Australian study, when in-hospital hip fractures were compared to those that occur in the community, a two-fold increase in hospital mortality, a greater need for referral to social and health centers at discharge and a much lower percentage of recovery of pre-fracture activities of life has been reported [21]. In a European study, Foss et al [22] included 44 consecutive patients with in-hospital hip fracture, a figure equivalent to 7% of all in-hospital fractures; these patients had a worse functional level and more comorbidities before the fracture and a significantly worse postoperative evolution, doubling the hospital stay and mortality during admission. It should be noted that half of the patients in this series had a previous history of falls and 75% of the patients suffered the fracture in the two first weeks of admission to an acute care unit. It can be concluded that this phase of admission should be targeted for reinforced fall prevention measures.

In our study, frequent risk factors for hospital hip fracture were the presence of dementia, confusional syndrome and malnutrition. Interventions designed to reduce the predisposing factors of delusion and confusion have been shown to be useful. Maracantonio et al [23], using a geriatric survey model in surgical patients with hip fracture, were able to reduce the incidence of delusion by almost 30%. With respect to malnutrition, other authors have previously considered the utility of a nutritional intervention in patients admitted with hip fracture, indicating that this helps to reduce the total number of days with delusion, the occurrence of pressure sores and hospitalization time [24]. In this study individuals living in institutions were almost two times more likely to sustain a hip fracture than those living in private homes. Institutionalized older people have increased prevalence of chronic illness, cognitive disorders, impairments of vision, strength and neuromuscular functioning and are frequently on polymedication [25-27]. Such individuals may be less exposed to the sun than those living in private homes and may have lower levels of vitamin D, thus placing them at increased risk of osteoporosis and possible muscle weakness [28].

The characteristics of the data base limit the clinical information to events happening only during admission. This is a limitation of the study as we ignore evolution time of diseases and risk factors and thus cannot establish a temporal relationship between hip fracture and this information. In other cases information related to hip fracture may not have been recorded in an effort to protect the reputation of physicians, other healthcare workers or the hospital center, or simply because the description of the complication is not very specific and cannot be properly interpreted. Nevertheless, given the clinical importance of hip fracture, it is unlikely that it will be omitted from the hospital discharge report. Another noteworthy limitation is that the administrative database did not include the treatments received by patients during admission. Falls during hospitalization are not recorded in the data base although it is a well known fact their direct association with fractures.

The results of our study suggest that patients admitted to internal medicine wards, showing some of the risk factors which we have pointed out for hip fracture such as very old age, female sex, admission from residence, the presence of malnutrition, delirium and dementia, should be identified early during admission in order to implement programmes directed to the prevention of falls and hip fractures. Even with the aforementioned limitations, the results of our study suggest that patients admitted to medical services with some of the risk factors identified should prompt the implementation of an individual programme of early detection of falls during admission. Fall prevention requires multidisciplinary strategies, which should first include adequate screening of patients to identify those at risk, a suitable protocol for the prevention of falls during admission, early intervention of delusional syndrome and proper drug prescriptions in these patients. Structural measures, such as changes in room and bath facilities, adjusted bed height, antislip stockings, appropriate lightning, pressure mats, and hip protectors, changes in room and bath furnishings, should also be implemented. Finally it is important to train nursing and medical personnel to recognize the risk factors for this complication [29,30].

## Conclusion

In the present study we report the incidence of hip fractures during hospital admissions to Spanish internal medicine services, which was almost 6 times higher than in patients who underwent surgery. In-hospital hip fracture notably increased mortality during admission, doubling the mean stay and mean cost of admission. For that reason, we stress the importance of designing and applying multidisciplinary plans capable to reduce the incidence of hip fractures in hospitalized patients.

## Competing interests

The authors declare that they have no competing interests.

## Authors’ contributions

RB: had the idea of this paper, she did all the statistical analysis and work with the results. AZ is the coordinator of the group and, as president of the Group of Medical Management of the SEMI (Sociedad Española de Medicina Interna – Spanish Society of Internal Medicine) was the official liaison with the Spanish Ministry of Health for the procurement of the crude data. JM confectioned the first draft and its final version for the approval of the other authors. SP help to obtain some meaning from the massive amount of results obtained from the raw data. JEL did all the pertinent crosses between partial results in order to achieved final results with statistical significance. JC helped write the discussion of the paper and made the bibliographic revision. JSR did the bibliographic revision. All authors contributed to the design and conduct of the study and read and approved the final manuscript.

## Pre-publication history

The pre-publication history for this paper can be accessed here:

http://www.biomedcentral.com/1471-2474/14/15/prepub
